# Novel genes upregulated when NOTCH signalling is disrupted during hypothalamic development

**DOI:** 10.1186/1749-8104-8-25

**Published:** 2013-12-23

**Authors:** Leslie Ratié, Michelle Ware, Frédérique Barloy-Hubler, Hélène Romé, Isabelle Gicquel, Christèle Dubourg, Véronique David, Valérie Dupé

**Affiliations:** 1 Institut de Génétique et Développement de Rennes, CNRS UMR6290, Université de Rennes 1, IFR140 GFAS, Faculté de Médecine, Rennes, France; 2 Amadeus Platform, SFR BIOSIT, Rennes, France; 3 Laboratoire de Génétique Moléculaire, CHU Pontchaillou, Rennes, France

**Keywords:** Notch, Proneural genes, Hypothalamus, Neuronal differentiation, Embryonic brain

## Abstract

**Background:**

The generation of diverse neuronal types and subtypes from multipotent progenitors during development is crucial for assembling functional neural circuits in the adult central nervous system. It is well known that the Notch signalling pathway through the inhibition of proneural genes is a key regulator of neurogenesis in the vertebrate central nervous system. However, the role of Notch during hypothalamus formation along with its downstream effectors remains poorly defined.

**Results:**

Here, we have transiently blocked Notch activity in chick embryos and used global gene expression analysis to provide evidence that Notch signalling modulates the generation of neurons in the early developing hypothalamus by lateral inhibition. Most importantly, we have taken advantage of this model to identify novel targets of Notch signalling, such as *Tagln3* and *Chga,* which were expressed in hypothalamic neuronal nuclei.

**Conclusions:**

These data give essential advances into the early generation of neurons in the hypothalamus. We demonstrate that inhibition of Notch signalling during early development of the hypothalamus enhances expression of several new markers. These genes must be considered as important new targets of the Notch/proneural network.

## Background

The hypothalamus influences a broad spectrum of physiological functions, including autonomic nervous system, reproduction and behaviour. Despite its physiological importance, we are only beginning to understand the molecular mechanisms underlying neural differentiation within this brain region. In the developing hypothalamus, progenitor domains were characterized by complex patterns of transcription factor gene expression, and an important yet unresolved question concerns the molecular determinants of the neurons produced in each progenitor domain [1,2].

The hypothalamus develops from the ventral region of the diencephalon and pattern formation depends on the activities of major protein signalling pathways, such as Sonic hedgehog (Shh) and bone morphogenic protein (BMP) [3]. They are involved in patterning, regional identity and cell fate determination. Proper development of the hypothalamic axis then requires signals, which lead to numerous types of neurons and glia. The temporal control of the transition from cell proliferation to cell differentiation in the nervous system is believed to involve many antagonist factors and a proper balance of their actions is essential for neurogenesis to take place [2,4].

It has been well described that Notch signalling is an evolutionary conserved mechanism that plays an essential role in maintaining neural progenitor identity and suppressing neuronal differentiation [5,6]. The transmembrane Notch receptor is activated upon binding to membrane-bound DELTA or SERRATE ligands present in adjacent cells. Upon activation of Notch signalling, the Notch intracellular domain (NICD) is released and forms a complex with the DNA-binding transcription factor RBPJ. This complex induces the transcription of repressor-type basic helix-loop-helix (bHLH) *Hes* and *Hey* genes by binding to their promoters [7,8]. Gain of function studies have revealed that constitutive Notch signalling leads to cells remaining as progenitors [9,10], whereas loss of NOTCH1 results in the premature differentiation of neurons at the expense of undifferentiated cells in the cerebellum [11]. Similarly, *Hes1* and *Hes3* double null mice show premature neuron formation in the mesencephalon and rhombencephalon [12]. Numerous studies have shown that this premature differentiation of neurons occurs through transient and sequential upregulation of proneural bHLH transcription factor genes [13-16]. From these studies and numerous others it has been proposed that to maintain neural progenitor cells a regulatory loop takes place between neighbouring cells. This loop involves the upregulation of Delta-ligand expression by proneural genes and downregulation of proneural gene expression by the Notch signalling pathway through the repressor *Hes/Hey* genes. This process is called lateral inhibition [13,17]. Thus, in the absence of *Hes* and *Hey* bHLH repressors, proneural genes such as *Ascl1* or *NeuroG* are significantly upregulated, and induce expression of a wide spectrum of neuron-specific genes leading to premature formation of early-born neurons [18].

Recently, Notch signalling has been strongly implicated in the differentiation of the mouse hypothalamic arcuate neurons (Arc) through a loss of function study in the mouse [16]. This study shows that Notch signalling affects maintenance of the hypothalamic neuronal progenitor pool by repressing the proneural gene, *Ascl1*. However, little is known about the molecular targets of this Notch/proneural network during this process.

In order to address the extent to which Notch signalling is required for functional neuronal development we have taken advantage of its function in the developing hypothalamus to characterize new target genes. A chemical approach was used to inactivate Notch signalling in the chick embryo in a specific time-window corresponding to the early steps of hypothalamic neurogenesis. Microarray analysis has allowed us to describe new critical neuronal precursor-specific markers that may be expressed under the control of the Notch/proneural network.

## Results

### Expression of Notch components during hypothalamus development

**Figure 1:**
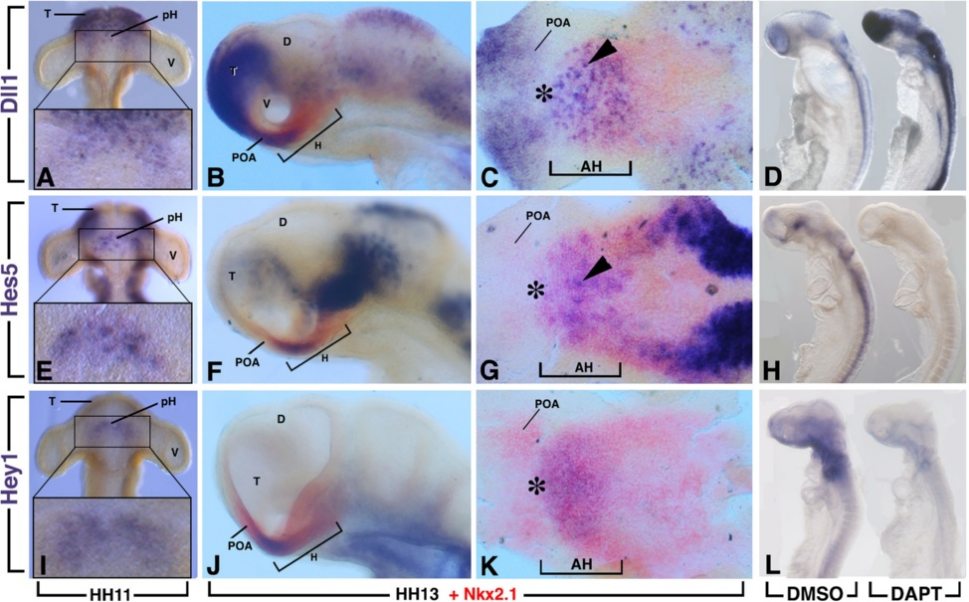
**Regionalized expression of the components of the Notch pathway in the hypothalamus.** Expression of *Dll1*, *Hes5* and *Hey1* at Hamburger and Hamilton (HH)11 and HH13 as indicated. All genes were detected initially as a crescent region between the two optic vesicles (boxes in **A**,**E**,**I**). **(B,C,F,G,J,K)** Double expression of Dll1, Hey1 or Hes5 (blue) with Nkx2.1 (red) at HH13, showing that the expression of all the genes occupies the rostral region of the hypothalamus. **(C,G,K)** Hemisected, flatmounted preparation of the ventral diencephalon of the corresponding embryos in **B**,**F**,**J**, respectively, with the optic vesicles removed and viewed from the ventral side. Arrowheads indicate scattered cell expression. Asterisk indicates rostral expression caudal to the prospective chiasmatic area. **(D,L)** HH10 chick embryos were treated with N-[3.5-difluorophenacetyl-L-alanyl)]-S-phenylglycine t-butyl ester (DAPT) or (DMSO) for 16 hours. **(H)** Embryos were treated for only 3 hours from HH12. There was downregulation of *Hes5***(H)** and *Hey1***(L)** expression after DAPT treatment, whereas Dll1 was upregulated **(D)**. AH, anterior (rostral) hypothalamus; D, diencephalon; H, hypothalamus; pH, hypothalamus primordium; POA, preoptic area; T, telencephalon, V, optic vesicle.

To identify the exact time point when the Notch pathway may be required during hypothalamus development, we examined the temporal expression of several Notch components in the developing chick brain between Hamburger and Hamilton (HH)8 and HH16 (Figure 1 and data not shown).

We observed that expression of *Dll1, Hes5* and *Hey1* were first detected in and around the ventral midline of the diencephalon just before HH11 with only a few marked cells labelled (Figure 1). At HH11, *Dll1* expression was found from the telencephalon to the rostral region of the diencephalon in scattered cells (Figure 1A). At this stage, *Hes5* was similarly expressed in the rostral region of the head except at the level of the most anteromedial part of the telencephalon where its transcripts were not found (Figure 1E). In contrast, *Hey1* expression was restricted to the rostroventral diencephalon between the two developing optic vesicles (Figure 1I). Importantly, ventral views of HH11 dissected neural tube revealed the ventral neurectodermal surface with similar expression patterns in a crescent-shaped area for *Dll1*, *Hes5* and *Hey1* centred around the midline between the optic vesicles (boxes in Figure 1A,E,I).

As development proceeds, the hypothalamus primordium was morphologically evident from approximately HH13. At this stage, double *in situ* hybridization with *Nkx2.1*, a general early marker of the hypothalamic plate [19], revealed an overlapping expression with *Dll1*, *Hes5* and *Hey1* that was restricted to the rostral region of the hypothalamus with rostral expression caudal to the prospective chiasmatic area (Figure 1C,G,K, asterisk). At this stage, *Dll1* displayed a fine salt-and-pepper-like pattern (Figure 1C, arrowhead). From HH13, *Nkx2.1* had just started to be expressed separately in the preoptic area of the basal telencephalon. *Dll1* was also expressed in the preoptic area but not *Hes5* and *Hey1. Dll1* and *Hes5* expression were also found overlapping with *Nkx2.1* in the lateral domain of the hypothalamic region.

**Figure 2:**
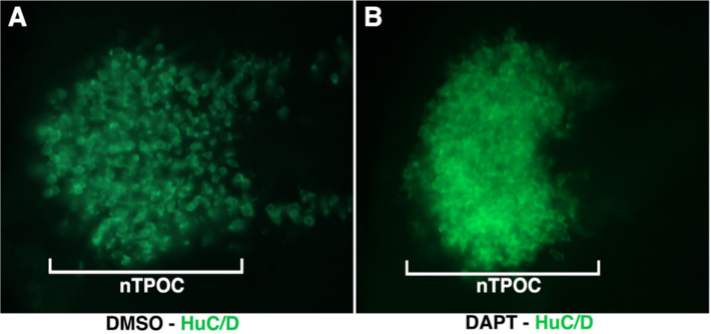
**Immunostaining for HuC/D in the rostral hypothalamus of DMSO and DAPT-treated chick embryos (n = 6). (A,B)** High magnification of hemisected, flatmounted preparations of the rostral hypothalamus at Hamburger and Hamilton (HH)13. The nucleus of the tract of the postoptic commissure neurons (nTPOC) was characterized by the expression of the Hu class of RNA-binding proteins that marks post-mitotic neurons. While the neurons were scattered in the DMSO control **(A)**, after DAPT treatment the nTPOC population becomes denser **(B)**. DMSO; DAPT, N-[3.5-difluorophenacetyl-L-alanyl)]-S-phenylglycine t-butyl ester.

The rostral hypothalamus gives rise to the nucleus of the tract of the postoptic commissure (nTPOC) at HH13 [20] as shown with the HuC/D-positive cells (Figure 2A). The members of the Notch signalling pathway that were expressed within the chick diencephalon from HH11 (Figure 1) mapped to the rostral end of the hypothalamus primordium corresponding to the nTPOC. The specific colocalisation of Notch components with the nTPOC at this stage underlines a strong contribution of this pathway during differentiation of hypothalamic neurons.

### Setting up an experimental strategy to identify Notch response genes in the early developing hypothalamus

To identify Notch response genes we used a chemical inhibition strategy combined with a global measure of transcript levels using chicken microarrays. The γ-secretase inhibitor N-[3.5-difluorophenacetyl-L-alanyl)]-S-phenylglycine t-butyl ester (DAPT) provides a powerful tool to inhibit cell differentiation processes regulated by Notch [14]. This treatment caused a rapid downregulation of *Hes5* expression after only 3 hours of DAPT-treatment (Figure 1H).

Based on our previous observations, showing that Notch components were first detected between HH10 and HH11 in the hypothalamus primordium, the genetic response to an inhibition of the Notch pathway was analysed from HH10 to HH13, corresponding to an overnight culture (16 hours). HH10 embryos were dissected and transferred into roller tubes, in which they were cultured for 16 hours either in Dimethyl sulfoxide (DMSO) or 40 μM DAPT-supplemented medium. With such conditions, the size of the embryos was similar between DAPT-treated and control embryos with no obvious morphological defects. In all DAPT-treated embryos analysed, the expression of *Hes5* (n = 20) and *Hey1* (n = 15) was dramatically reduced or abolished in the neural tube and forebrain, including the ventral diencephalon (Figure 1L). It has been well established that downregulation of *Hes5* de-represses the expression of *Dll1*[18]. Accordingly, the DAPT treated embryos (n = 15) exhibit increased expression of Dll1 after 16 hours of treatment (Figure 1D). Furthermore, Notch inhibition during this period was associated with an increase in the number of neurons and a loss of the scattered neurons in the rostral hypothalamus (Figure 2). Therefore, a treatment starting at HH10 for 16 hours appeared to be appropriate to identify direct or indirect downstream targets of Notch signalling during the first step of hypothalamic neurogenesis.

### Identification of Notch response genes in the developing forebrain

HH10 embryos were treated with DAPT and harvested after 16 hours culture, at approximately HH13, for a comparative microarray analysis. To restrict our findings to genes that were controlled by Notch in the prosencephalon-forming region, we dissected a domain that was situated rostral to the mesencephalic region containing the hypothalamic primordium. This domain was chosen because it was morphologically well delimited at the stage of dissection. For each experiment, five prosencephalons from the same culture were dissected and pooled to obtain total RNA. Four different sets of experiments were collected and tested by microarray, using chick genome 4x44k DNA-microarray (Agilent, Massy, France; [21]). The DMSO and DAPT-treated embryos were directly compared by Genespring GX software version 12.0 Agilent. We opted for a fold-change (FC) of 1.3 cut-off and identified 789 downregulated and 769 upregulated genes in the DAPT-treated forebrain.

It was noted that members of the Notch signalling pathway were significantly enriched in Gene Ontology (GO) term analysis. As expected, many Notch transcriptional targets and/or Notch pathway components have their expression consistently modified in our data (Table 1). The direct Notch target gene *Hes5* was highly downregulated (FC −56.83); also downregulated was *Hey1* (FC −2.89). As expected, *Dll1* was upregulated (FC 2.26) [22]. Additionally, we identified changes in expression levels of other components of the Notch pathway: *Jag1* (FC −1.58) [23], *Numb* (FC –1.49) [24], *Lfng* (FC −1.45), *Notch1* (FC −1.31) and *Nrarp* (FC −2.01) [25] that were downregulated (Table 1). Other components were upregulated, which have not been previously described as Notch targets: *Aph1A* (FC 1.34) and *Mfng* (FC 3.14) [26].

**Table 1:** Example of expression values for genes selected with an absolute fold change ≥1.3 after Gene Ontology enrichment

**Gene symbol**	**Accession number**	**Description**	**Fold change value**
*Notch signalling pathway components*			
*Hes5*	NM_001012695	Hairy and enhancer of split 5 (*Drosophila*)	−56.83
*Hey1*	XM_425926	Hairy/enhancer-of-split related with YRPW motif 1	−2.89
*Nrarp*	XM_428951	Notch-regulated ankyrin repeat protein	−2.02
*Jag1*	XM_415035	Jagged 1	−1.58
*Numb*	NM_204835	Numb homolog (*Drosophila*)	−1.49
*Lfng*	NM_204948	LFNG O-fucosylpeptide 3-beta-N-acetylglucosaminyltransferase	−1.46
*Notch1*	XM_415420	Notch 1	−1.31
*Notch2*	XM_001233595	Notch 2	−1.30
*Aph1a*	XM_001233301	Anterior pharynx defective 1 homolog A *(C. elegans)*	1.34
*Dll1*	NM_204973	Delta-like 1 (Drosophila)	2.26
*Mfng*	XM_416278	MFNG O-fucosylpeptide 3-beta-N-acetylglucosaminyltransferase	3.15
*Nervous system development Gene Onotology term* (*upregulated genes*)			
*Neurog1*	NM_204883	Neurogenin 1	8.85
*Nhlh2*	AF123885	Nescient helix loop helix 2	4.18
*Nefm*	NM_001101730	Neurofilament, medium polypeptide	4.05
*Tagln3*	XM_416634	Transgelin 3	3.69
*Nhlh1*	NM_204121	Nescient helix loop helix 1	3.47
*Stmn2*	NM_205181	Stathmin-like 2	2.49
*Nr5a1*	NM_205077	Nuclear receptor subfamily 5, group A, member 1	1.87
*Nrg1*	NM_204117	Neuregulin 1	1.77
*Chga*	CR523039	Chromogranin A (parathyroid secretory protein 1)	1.74
*Sim1*	U40058	Single-minded homolog 1 (Drosophila)	1.74
*Bsx*	NM_204512	Brain-specific homeobox	1.68
*Ascl1*	NM_204412	Achaete-scute complex homolog 1 (Drosophila)	1.67
*Nr5a2*	NM_205078	Nuclear receptor subfamily 5, group A, member 2	1.59
*Gap43*	XM_425527	Growth associated protein 43	1.57
*Neurog2*	NM_204796	Neurogenin 2	1.57
*Sox14*	NM_204761	SRY (sex determining region Y)-box 14	1.51
*Robo2*	XM_416674	Roundabout, axon guidance receptor, homolog 2 (Drosophila)	1.46
*Cntn2*	NM_001004395	Contactin 2 (axonal)	1.39
*Slit1*	XM_421715	Slit homolog 1 (Drosophila)	1.37
*Hes6*	BX935736	Hairy and enhancer of split 6 (Drosophila)	1.36
*Bdnf*	NM_001031616	Brain-derived neurotrophic factor	1.34
*Foxn4*	NM_001083359	Forkhead box N4	1.31
*Chrdl1*	NM_204171	Chordin-like 1	1.30

HH10 embryos were treated with N-[3.5-difluorophenacetyl-L-alanyl)]-S-phenylglycine t-butyl ester (DAPT) or Dimethyl sulfoxide (DMSO) for 16 hours, and global change in gene expression of the forebrain was compared by microarray analysis. Two Gene Ontology enriched terms have been selected: *Notch signalling pathway* and *Nervous system development*. This Gene Ontology term contains 271 enriched genes; 135 were upregulated. Here we have listed the 23 genes for which we have obtained RNA probes. The title and description for each gene are given as well as the accession number and the fold change.

Enrichment for the GO term *Nervous System Development* that included 271 genes was observed. Several proneural genes normally repressed by the Notch pathway were upregulated [17]. These included *Ascl1* (FC 1.67), *NeuroG1* (FC 8.85) and *NeuroG2* (FC 1.57). Thus, DAPT treatment caused the expected response amongst Notch signalling pathway components, including Notch effector genes and proneural bHLH transcription factors. This demonstrated the validity of the microarray approach for identifying new target genes of the Notch signalling pathway.

### *In situ* hybridization validation of upregulated genes

To identify new molecular markers regulated directly or indirectly by Notch signalling we focused our efforts on the upregulated genes. We obtained efficient RNA probes for 23 upregulated markers from the enriched GO term *Nervous system development* (Table 1). These genes represented diverse functional classes and were either uncharacterized or only partially described during hypothalamus development. Some of these genes were already known hypothalamic markers (such as *Robo2* and *Slit1*) or Notch targets in other tissues (such as *Nhlh1* and *Stmn2*) [1,2,27]. Most of the selected genes consisted of transcription factors, binding proteins or specific neural progenitor genes.

**Figure 3:**
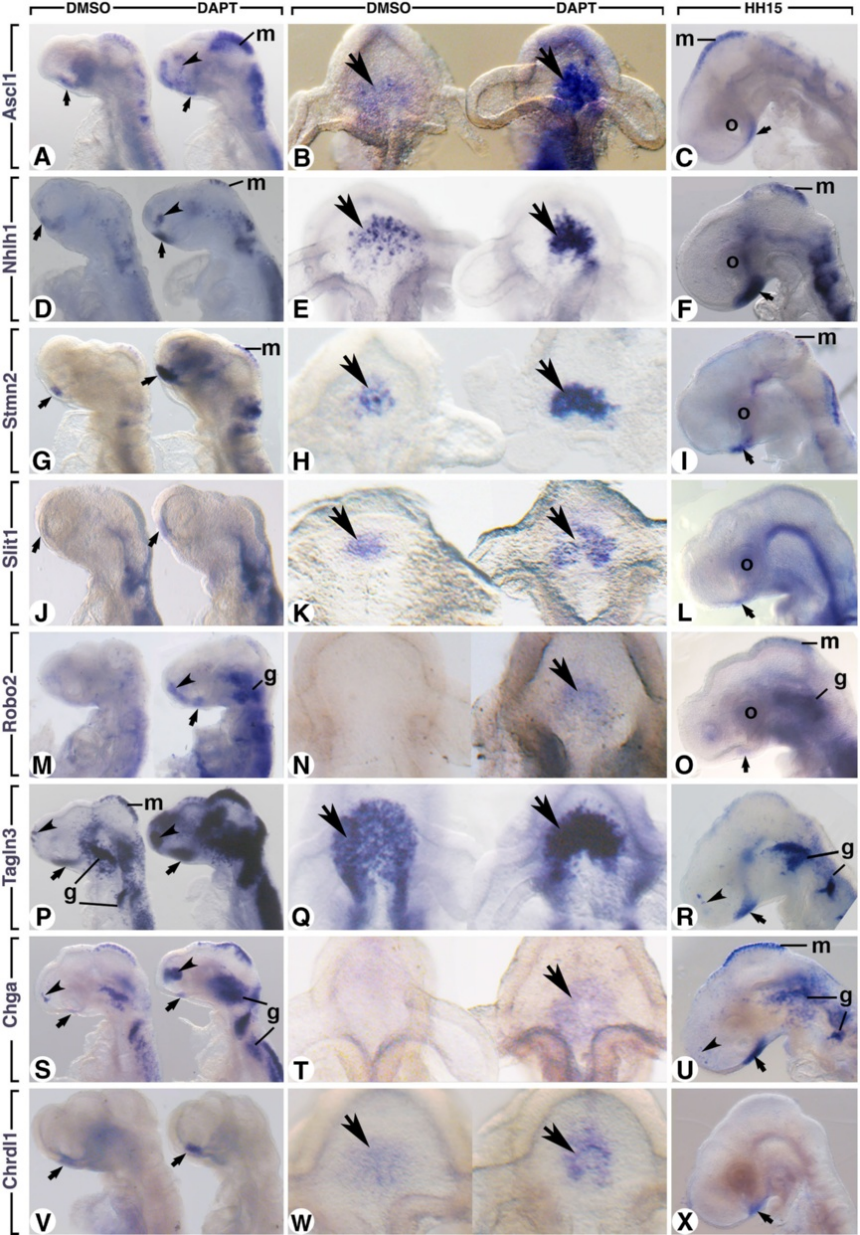
**Regionalized expression of upregulated genes in the hypothalamus in DAPT-treated chick embryos.** Whole embryo *in situ* hybridization was performed with *Ascl1***(A-C)**, *Nhlh1***(D-F)**, *Stmn2***(G-I)**, *Slit1***(J-L)**, *Robo2***(M-O)**, *Tagln3***(P-R)**, *Chga***(S-U)** and *Chrdl1***(V-X)** riboprobes on control and DAPT-treated embryos after 16 hours ex-ovo culture and on Hamburger and Hamilton (HH)15 embryos as indicated on the figure. In **B**, **E**, **H**, **K**, **N**, **Q**, **T** and **W**, mesenchymal cells and surface ectoderm were removed to obtain dissected neural tubes (ventral view) from the embryo described on the left side. Small arrows highlight specific expression of all these probes in the rostral hypothalamus. Arrowheads point to the expression in the olfactory epithelium. g, ganglia; m, roof of the mesencephalon; o, optic vesicles. DAPT, N-[3.5-difluorophenacetyl-L-alanyl)]-S-phenylglycine t-butyl ester.

We systematically compared the expression of these genes in control (DMSO) to DAPT-treated embryos in the same conditions as the microarray (Figure 3). Remarkably, among the upregulated genes tested, eight displayed a prominent tightly restricted expression in the rostral hypothalamus in DAPT-treated embryos (Figure 3). Interestingly, for some of these genes, this upregulation was not just restricted to the hypothalamus but was also within other domains of expression such as the roof of the mesencephalon, the olfactory epithelium or the forming ganglions.

After DAPT-treatment, *Ascl1* expression (n = 10) was upregulated in all tissues of the head that normally expressed this gene (Figure 3A,B). This included the neuroectoderm of the ventral diencephalon corresponding to the developing hypothalamus as shown with the dissected neural tube (Figure 3B). Expression of *Nhlh1* has been shown to be regulated by *Ascl1* and previously described in the olfactory epithelium, cranial ganglia and dorsal root ganglia [13] but not in the developing hypothalamus. Here, scattered *Nhlh1*-positive cells were found throughout the ventral midline between the two optic vesicles of HH13 control embryos (Figure 3E). In contrast, the density of *Nhlh1*-positive cells appeared higher in DAPT embryos (n = 12) at the level of the ventral diencephalon (Figure 3D,E). Furthermore, *Nhlh1* was aberrantly expressed in the olfactory placodes and the roof of the mesencephalon at this stage, whereas only a few scattered cells expressed this gene in the control embryos. Importantly, *Ascl1* and *Nhlh1* displayed the same profile of upregulation in DAPT-treated embryos. *Stmn2* (previously called SCG10) codes for a cytoskeletal protein and is an early marker of neuronal phenotype [28]. Here, in DAPT embryos (n = 10) this gene was strongly upregulated in the developing hypothalamus, and its scattered distribution was also lost (Figure 3G,H). This upregulation has been classically associated with upregulation of *Ascl1* and *Nhlh1*[13]. Altogether this signifies a change in the number and density of neurons and a precocious neurogenesis in and around the ventral midline. This was also confirmed using the HuC/D antibody on DAPT-treated embryos (Figure 2B). These observed effects fit with the expected role of Notch in regulating the number of neuronal precursors by lateral inhibition [17,29].

Among the other upregulated genes in the ventral diencephalon, two have been previously described in the mouse hypothalamus: *Robo2* and *Slit1*[27]. In the control embryos, *Slit1* expression was just emerging, whereas after DAPT treatment (n = 13), *Slit1* was upregulated in the ventral midline area (Figure 3J,K). At the same stage, *Robo2* expression was not detectable in the ventral midline of the control embryos, whereas DAPT-treated embryos (n = 14) expressed this gene (Figure 3M,N). *Robo2* was also upregulated in the forming ganglions and olfactory placodes when Notch signalling was inhibited (Figure 3M). Notably, all these tissues normally displayed Notch activity [29].

Most interestingly, *Tagln3*, *Chga* and *Chrdl1* were three genes that were not well documented. Their expressions have never been described in the developing hypothalamus or dependent of Notch signalling.

TAGLN3, a microtubule-associated protein, has been described as a neuron-specific protein in the developing neural tube [30]. In the control brain (n = 17), *Tagln3* was strongly expressed in the ventral diencephalon and showed a scattered mRNA distribution, which was lost after DAPT treatment (Figure 3P,Q). *Tagln3* was upregulated in all the tissues normally expressing this gene, which included the olfactory placodes, the ventral diencephalon, the mesencephalic roof, the ganglions and the neural tube (Figure 3R). *Chga* has been shown to be a member of the granin family of neuroendocrine secretory proteins, located in secretory vesicles of neurons and endocrine cells [31]; however, little has been discussed about its role during the early embryonic stages. When treated with DAPT, the level of *Chga* expression was increased in all the tissues normally expressing this gene at HH13 (n = 19), which includes the ventral diencephalon and the olfactory placodes as for *Tagln3* (Figure 3S). *Robo2* and *Chga* were expressed in the hypothalamus at HH13 but only when Notch activity was inhibited (HH13 in Figure 3N,T). Nevertheless, when Notch activity was normal these genes were expressed in the ventral hypothalamus from HH15 (HH15 in Figure 3O,U).

Notably, at HH15, *Tagln3* and *Chga* were similarly expressed in and around the ventral midline of the diencephalon, the forming ganglions and dorsal mesencephalon (Figure 3R,U). Interestingly, a few scattered cells expressed these genes in the olfactory epithelium (Figure 3R,U, arrowheads) suggesting a function for these new markers during olfactory sensory neurons differentiation.

Remarkably*, Chrdl1*, a BMP antagonist [32], had its expression restricted to the developing hypothalamus at HH13 and in DAPT treated embryos (n = 20). *Chrdl1* expression was clearly upregulated in this area (Figure 3V,W). At HH15, *Chrdl1* kept its restricted expression in the rostroventral hypothalamus and expression was not detected in other tissues of HH15 embryos (Figure 3X and data not shown).

**Figure 4:**
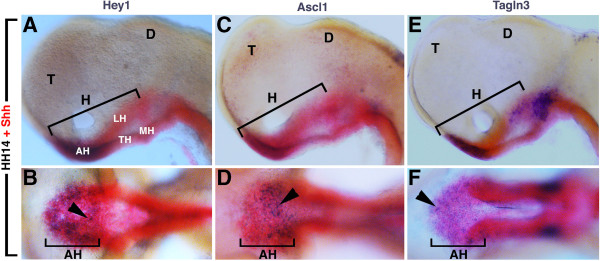
**(A-F) Hamburger and Hamilton (HH)14, dissected chick neural tube.** Expression of Notch pathway component, *Hey1***(A,B)**, the proneural gene *Ascl1***(C,D)** and a new putative target gene, *Tagln3***(E,F)** in the hypothalamus compared to *Shh* expression at HH14. The figure shows that these three genes were similarly expressed in the rostral region of the hypothalamus **(AH)**. **(A,C,E)** Lateral views with the rostral region on the left. **(B,D,F)** Ventral views of the corresponding embryos **A**, **C**, and **E**. Arrowheads show the patched expression of *Hey1*, *Ascl1* and *Tagln3*. AH, anterior (rostral) hypothalamus; D, diencephalon; H, hypothalamus; LH, lateral hypothalamus, MH, mammillary region of the hypothalamus, T, telencephalon, TH, tuberal region of the hypothalamus.

All eight genes were visibly expressed ventrally at the level of the midline in the area localized between the two optic vesicles (Figure 3) corresponding to the expression domain of the Notch components previously described in Figure 1 and the nTPOC (Figure 2). In order to confirm that these expression domains were restricted to the future rostral hypothalamus, in addition to *Nkx2.1*, *Shh* was also used as a marker. At HH14, *Shh* was expressed exclusively along the ventral midline of the neural tube but, contrary to *Nkx2.1*, displayed a dynamic distribution at the level of the hypothalamus subregions [33]. As shown by the red staining in Figure 4, the future hypothalamus was arranged rostrocaudally into the anterior (rostral), tuberal and mammillary region (Figure 4). To compare precisely the expression profiles of *Shh* and *Hey1*, embryos were double labelled at HH14. Expression of *Hey1* was confined to the *Shh*-positive cells of the anterior hypothalamus in the same region that the proneural gene *Ascl1* and *Tagln3* were expressed (Figure 4). At this stage, *Hey1*, *Ascl1* and *Tagln3* displayed a salt-and-pepper-like pattern in this area.

### Identification of a transcriptional program regulated *by Hes5/Hey1*, *Ascl1* and *Nhlh1*

It has been established that a conserved Ascl1/Dll1/Hes/Hey/Nhlh1 molecular circuitry operates within the progenitor pool to coordinate neurogenesis [34]. In order to test if the new candidate genes characterized in this study could have their expression directly regulated by this network, an *in silico* approach was used to determine DNA-binding signatures for ASCL1, HES5, HEY1 and NHLH1 and to detect the presence or the absence of these sites at the level of the promoter of the genes implicated in this circuitry.

**Figure 5:**
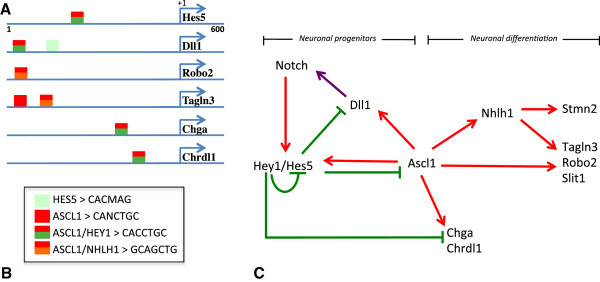
**Summary of potential regulatory interaction model of gene regulation for neurogenesis in the hypothalamus revealed by *in silico *analysis and literature. (A)** Schematic of mouse *Hes5*, *Dll1*, *Robo2*, *Tagln3*, *Chga* and *Chrdl1* promoters showing the position of evolutionarily conserved binding motifs for HES5 (light green box), ASCL1 (red box), antagonist binding sites ASCL1/HEY1 (MHAM, red and green box) and ASCL1/NHLH1 (MNAM, red and orange box). Regulatory regions comprise 500 bp before to 100 bp after the transcription start site (+1, blue arrow). The schematic represents only the motifs that were conserved among several species. **(B)** Consensus chick and mouse box sequences from the literature and this study. **(C)** A model of interaction between the negative regulators HES5 and HEY1 and the positive regulators ASCL1 and NHLH1 based on the literature and present data. Activations are identified with a red arrow and repressions are identified with a green barred line. The purple arrow refers to the activation of NOTCH receptor by its ligand DLL1. (Bertrand, 2002; Gohkle, 2008, Castro, 2011, de la Pompa 1997, and present data) [13,29,34,50].

By using the MatInspector (Genomatix, Munich, Germany) and Evolutionary Conserved Region (NIH, USA) browser software, the available proximal promoter sequences of selected genes were screened for binding sites in numerous organisms in order to find some cross-species genomic conservation. These promoters were defined as sequences 0.5 Mb before and 0.1 Mb after the transcription start site (+1). Despite a poor degree of nucleotide homology between mouse and chick proximal promoter regions of *Hes5* and *Dll1*, we found a conserved sequence of seven nucleotides (5’-CACCTGC-3’ - MHAM) corresponding to an antagonist-binding site for ASCL1/HEY (Figure 4). Importantly, this motif allowed us to give a better stringency condition to the *in silico* study compared to degenerated E-box (5'-CANNTG-3') and N-box (5’-CACNAG-3’) sequences attributed to ASCL1 or HEY1 alone [13]. Here, we described this motif at the level of the *Chga* and *Chrdl1* proximal promoters (Figure 5). This ASCL1/HEY motif was also found at the level of the *Mnfg* promoter, a known direct target of ASCL1 [34].

We also observed another conserved sequence of strict 7-mer nucleotides (5’-GCAGCTG-3' - MNAM) previously described as a direct binding sequence for Ascl1 [35] that correspond to an antagonist-binding site for ASCL1/NHLH1. This sequence was found conserved in the proximal promoter region of *Robo2* and *Tagln3*.

It can therefore be proposed that these regulatory elements, which were selected for enrichment in the Ascl1/Dll1/Hes/Hey/Nhlh1 molecular circuitry, may represent signatures to drive expression in some neural tissues. Obviously the functional relevance of this finding has yet to be exhaustively explored. This *in silico* approach along with literature led us to propose a predictive model of gene regulation for neurogenesis in the hypothalamus, but this cannot be excluded as a model for other neuronal tissues such as the ganglia where neurons also differentiated during early development and expressed these new target genes (Figure 5).

## Discussion

In this report, *ex ovo* roller culture of chick embryos has allowed DAPT to be delivered at a specific concentration and time. We show that pharmacological inhibition of Notch signalling can phenocopy the experimental results obtained with other methods, but allows better temporal control [29]. Treatment with DAPT caused a rapid decline in downstream components of the Notch signalling pathway (that is, *Hes5*) that initiates the molecular cascade leading to the upregulation of the proneural bHLH gene, *Ascl1*, and the Notch ligand, *Dll1*. Numerous studies using genetic models have reported the same effect at the level of various developing tissues such as the olfactory epithelium, the roof of the mesencephalon and neural tube [18,29,36-39]. An example is the phenotype of the null mutant mice for RBPJ, the essential transcription factor mediating signalling of all Notch receptors [29]. *Hes5* transcripts normally present in the brain and spinal cord were completely eliminated in RBPJ mutant embryos, whereas *Dll1* and *Ascl1* were upregulated along with neuronal differentiation markers such as *Nhlh1*[29]. In our *ex ovo* experiments, this premature neuronal differentiation was similarly observed in the neural tube, mesencephalon and developing cranial placodes and ganglia. This study using RBPJ null mice further highlights the validity of our method.

Surprisingly, an accurate description of Notch activity during the earliest stages of mouse or chick hypothalamus development when the first neurons differentiate was not reported. However, scarce studies indicate that *Dll1*, *Notch2* and *Hes5* expression was spatially and temporally restricted to the developing mouse diencephalon from E9.5 (+/− HH11 in chick) [4,40,41]. A temporal assessment of Notch pathway effectors (for example, *Hes5*) indicated high and localized expression within the chick diencephalon in the ventral-most cells as early as HH11. Based on the hypothalamus markers, *Nkx2.1 and Shh*[19,33], we concluded that this Notch-positive region corresponds to the hypothalamic cells of the prospective anterior hypothalamus. Because *Hes5* was transcribed in the embryo solely in response to Notch signalling [8], we thus demonstrate that the first evidence of Notch activity in the chick hypothalamus starts very early on during the neuron progenitor expansion [20]. The *Hey1* expression domain overlaps with *Hes5* in the presumptive hypothalamus, further suggesting that these genes may have overlapping functions in this tissue. These results were supported by a study that has shown that a reduction in *Hes/Hey* gene dosage led to an overproduction of neurons during hair cell formation [42].

In agreement with previous studies conducted in vertebrate embryos, the salt-and-pepper-like patterns observed for *Dll1, Hes5*, *Hey1* and *Ascl1* in the rostral hypothalamus suggested that lateral inhibition regulates early production of hypothalamic neurons [17,43]. During this event, scattered cells expressing *Dll1* activate Notch in their neighbouring cells inducing the expression of the transcriptional repressors of the *Hes*/*Hey* family genes. They repress the expression of proneural bHLH genes that are essential for specifying neural fate [17]. In this study, by treating chick embryos with DAPT from HH10, *HuC/D*-positive cells were found grouped in a dense cluster of adjacent cells in DAPT-treated embryos, whereas they remained scattered in control embryos. Thus, an excessive number of cells differentiated into neurons in the ventral hypothalamus in the absence of Notch activity. This precocious neurogenesis was the typical “neurogenic” phenotype expected for the loss of function of Notch if working by lateral inhibition [9,36]. This was confirmed by the upregulation of *Ascl1* and by the loss of the salt-and-pepper profile of the neuronal markers *Nhlh1* and *Stmn2* in the absence of Notch activity. Thus, we propose for the first time that a mechanism of lateral inhibition operates in the rostral hypothalamus of the vertebrate brain to generate neurons.

This mechanism was most probably mediated by the proneural gene *Ascl1*. At E10.5, *Ascl1* was the major bHLH proneural gene expressed at the level of the mouse basal plate expressing *Nkx2*.1, whereas other proneural genes such as *Neurog1* and *Neurog2* have their expression restricted to the dorsal brain [40]. In the chick brain, *Neurog1* and *Neurog2* expression was not detected during the early phase of ventral hypothalamus development (data not shown) whereas *Ascl1* expression was restricted to the rostral domain of *Shh*. Concordantly, targeted mutagenesis demonstrated an essential role for *Ascl1* during the generation of ventral neuroendocrine neurons as well as playing a central role in subtype specification of these neurons [40].

Loss of function through gene mutations provides an essential tool to confirm whether the Notch pathway has an early function during hypothalamus neurogenesis. However, single null mice for components of Notch (ligands or receptors) and their mediators (such as *Hes1*, *Hes5* or *Hey1*) display defects in restricted areas of the developing embryo [36,44-46]. While *Hes5* mutant mice do not show any gross abnormalities, in the absence of both *Hes1* and *Hes5* genes cell differentiation was severely accelerated, leading to aberrant neuronal localization [18]. This redundancy between the major genes of the Notch pathway may explain why a role for Notch during the earliest stages of hypothalamus formation has never been strongly attributed to this pathway before. Only recently, conditional knockout mice lacking RBPJ in the forming hypothalamus were obtained [16]. However, no investigations at the early stage of hypothalamus development have been done. Concordantly, these mice showed an increased proliferation of the Arc neurons at E13.5 that may contribute to the increased body weight phenotype observed in the mutants. This abnormality probably takes place at earlier stages, as the Nkx2.1-Cre that has been used to drive Notch gene inactivation was active as early as E10.5 [47].

Other major findings in this paper were the characterization of new components of the differentiation programme of neural progenitor cells of the hypothalamus but also of the ganglia and olfactory epithelium. We have independently verified the results of our microarray screen using RNA *in situ* hybridization and demonstrated that many of these genes were specifically expressed in the hypothalamus at HH15. The specific expression of *Chrdl1* in the rostral hypothalamus was very fascinating, as the BMP pathway has been strongly implicated, coupled with *Shh* for the early patterning of the hypothalamus and in neuronal maturation [3]. This suggests that Notch signalling may regulate the activity of BMP signalling in this area.

We have additionally identified markers of early differentiating neurons such as *Nhlh1*, markers implicated in axon outgrowth such as *Stmn2* and in axon guidance such as *Robo2* and *Slit1. Robo2* and *Slit1* were expressed in the mouse developing hypothalamus from E10.5 and were required to maintain proper balance between primary and intermediate neuronal progenitors [27]. This function was probably conserved in the chick.

Most interestingly, characterization of other unknown potential neuronal markers such as *Tagln3* and *Chga* in the developing hypothalamus gave us a high level of confidence in our approach to discover new molecules regulated by Notch signalling. TAGLN3 is a microtubule-associated protein [48] and CHGA is a protein released in the circulation by neurons that interacts with STMN2 in human neuroendocrine cells [49].

At HH15, the comparison between *Tagln3* and *Chga* expression revealed a virtually identical expression pattern, not only in the hypothalamus but also in the olfactory epithelium and forming ganglions. They were similarly upregulated in all these tissues when Notch activity was blocked. These expression patterns restricted to neural tissue have not been reported before. Therefore, these genes must be considered as important new molecular markers for neurogenesis.

Importantly, *Tagln3, Chga, Robo2, Slit1* and *Chrdl1* expression has been highlighted in this study to be controlled by the Notch/proneural genes network. This cascade of genes allows cells to escape lateral inhibition (neuronal progenitor) to enter the pathway that leads to terminal neuronal differentiation (Figure 5). It was clear that coordination between numerous proneural factors was necessary for the specification of the neuronal subtypes of the ventral hypothalamus. There are numerous studies in which *Ascl1* has been identified to regulate other bHLH genes (that is, *Neurog3*, *NeuroD*, *Nhlh1*), which in turn activates neuron-specific structural genes such as *Stmn2*[4,13]. Interestingly, a genome-wide chip assay has shown that ASCL1 directly binds *Tagln3* promoter [50]. ASCL1 and CHGA have the same overexpression profile in small-cell lung cancer [51]. Although we have no *in vitro* or *in vivo* evidence that HES5, HEY1, ASCL1 and/or NHLH1 directly binds to or regulates the expression of *Chga*, *Chrdl1* and *Robo2* genes, we have found several well conserved putative HEY1-, and ASCL1- and NHLH1-binding sequences located within the 600 bp around their transcription start sites. Certainly, a more detailed fate map study of these neuronal markers will be required to better understand how they contribute to the specification of the hypothalamic sensory territories. In the meantime, our data have been compared with other screens and genome wide chip experiments to build a model of the regulatory cascade [34,50] that may regulate neurogenesis at the level of the hypothalamus and possibly other regions of the developing embryo (Figure 5).

## Conclusions

Given the almost universal use of Notch/proneural network in cell fate renewal and transition, it was necessary to look for new molecular targets. We uncovered that Notch signalling modulates the generation of neurons in the early developing hypothalamus by lateral inhibition. Most importantly, our global approach allowed us to characterize several new markers expressed in this tissue and that may have their expression under the control of the Notch/proneural genes oscillating regulation. The elucidation of this Notch/proneural transcriptional cascade including these new genes will be a challenge in the future since hypothalamic development has considerable importance for human health. Genetic defects in the development of specific cell subtypes in hypothalamus have already been reported for congenital obesity [52].

## Methods

### Roller-tube culture and drug treatments

Isa Brown fertile hen eggs were obtained from the Amice-Soquet hatchery (Lanrelas, France) and incubated in a humidified 38°C incubator for the desired stages. Embryos were staged according to Hamburger and Hamilton [53]. Embryos were collected at HH10 and cultured as described previously [54]. The γ-secretase inhibitor DAPT (Sigma, France) was dissolved in DMSO [55]. Embryos were treated with DMSO or 40 μM of DAPT in L15 culture medium (supplemented with chick serum and gentamycin). Animal experimentation protocols conformed to the European Union guidelines (RL2010/63/EU). Ethical approval was not required.

### mRNA expression profiling on microarray

A whole genome microarray analysis was performed on total RNA extracted from 5-pooled prosencephalon. Chick embryonic prosencephalon was dissected after 16 hours roller culture with DMSO or DAPT. Total RNA was immediately extracted. The RNA quality had been controlled by 2100 Bioanalyzer (Agilent). Total RNA (50 μg) from each sample was reverse transcripted and cRNAs were prepared according to the Agilent recommendations and following the one-colour protocol to be labelled with Cy3-CTP. Hybridization was accomplished on the Agilent 4x44K whole chicken genome *in situ* oligonucleotide microarray (Agilent) [21]. Image acquisition was performed using the Agilent Scanner (Biogenouest, Rennes) and signals were extracted by Agilent Feature Extraction software. The array files were submitted to the NIH Gene Expression Omnibus database (accession number GEO: GSE53129). Data normalization was performed by a per-chip 50^th^ percentile method by the Genespring Agilent GX12. GO analysis was performed using Webgestalt online software (http://bioinfo.vanderbilt.edu/webgestalt/). The *Homo Sapiens* Genome was selected as a reference set for enrichment analysis. Webgestalt used a hypergeometric statistical method and an adjustment of Benjamini-Hochberg for the Multiple Test with a significance level of 0.05. *Nervous system development* enrichment was significant with adjP = 1.55e-22. *Notch signalling pathway* enrichments performed on KEGG and Pathways Commons analysis were significant with respect to: adjP = 6.96e-07 and adjP = 5.80e-06.

### Whole-mount *in situ* hybridization and immunohistochemistry

After harvesting, embryos were fixed with 4% paraformaldehyde in phosphate buffered saline. Whole-mount *in situ* hybridization was performed as described [56]. All probes, except *Dll1*, were generated by PCR which were subcloned in pCR®II-TOPO® (Invitrogen, Cergy Pontoise, France) and used to transcribe the antisense RNA probes. A plasmid carrying chick *Dll1* (NotI-T3; gift of Frank Schubert, UK) was used. Double *in situ* hybridization on whole embryos was done with Digoxigenin and Fluorescein labelled RNA probes (Roche, Meylan, France). The protocol for immunohistochemistry has been described previously [57]. Whole-mount roller culture embryos were stained using pro-neural antibody, anti-human HuC/D (1:500, 16A11, Life technologies, Saint-Aubin, France).

### Design of transcription factor binding sites and comparative genomic analysis of promoters in various species

Mouse and chick genomic sequences were downloaded from the UCSC Genome Browser and Entrez Gene Databases. Mouse promoters were downloaded from The Eukaryotic promoter database and compared to proximal promoters predicted with PromScan Software. Mouse and chick promoters were annotated with different specific motifs such as TATA boxes using YAPP Software (http://www.bioinformatics.org/yapp/cgi-bin/yapp_intro.cgi). Predictive transcription factor-binding consensus sequences were obtained using MatInsepctor Position Specific Scoring Matrix libraries [58]. Relevant binding sites were analysed using multiple alignments and consensus sequences were transformed in IUPAC strings. These strings were reported on selected gene promoters. Finally a comparative genomic study was performed for binding sites between mammal and bird when available.

## Abbreviations

Arc: Hypothalamic arcuate neurons; bHLH: Basic helix-loop-helix; BMP: Bone morphogenic protein; DAPT: N-[3.5-difluorophenacetyl-L-alanyl)]-S-phenylglycine t-butyl ester; DMSO: Dimethyl sulfoxide; FC: Fold change; GO: Gene Ontology; HH: Hamburger and Hamilton; NICD: Notch intracellular domain; nTPOC: Nucleus of the tract of the postoptic commissure; PCR: Polymerase chain reaction; Shh: Sonic hedgehog.

## Competing interests

The authors declare that they have no competing interests.

## Authors’ contributions

LR, VDa and VDu set up and designed the experiments. LR, MW, HR and IG performed the experiments. LR and FB-H performed the *in silico* approach. LR and CD performed the microarray analysis. LR, MW, and VDu wrote the manuscript, and all authors read discussed and edited the manuscript. All authors have read and approved the final manuscript.

## References

[B1] Kurrasch DM Cheung CC Lee FY Tran PV Hata K Ingraham HA The neonatal ventromedial hypothalamus transcriptome reveals novel markers with spatially distinct patterning J Neurosci 2007 8 13624 13634 10.1523/JNEUROSCI.2858-07.2007 18077674 PMC6673626

[B2] Shimogori T Lee DA Miranda-Angulo A Yang Y Wang H Jiang L Yoshida AC Kataoka A Mashiko H Avetisyan M Qi L Qian J Blackshaw S A genomic atlas of mouse hypothalamic development Nat Neurosci 2010 8 767 775 10.1038/nn.2545 20436479 PMC4067769

[B3] Ohyama K Das R Placzek M Temporal progression of hypothalamic patterning by a dual action of BMP Development 2008 8 3325 3331 10.1242/dev.027078 18787065

[B4] Pelling M Anthwal N McNay D Gradwohl G Leiter AB Guillemot F Ang SL Differential requirements for neurogenin 3 in the development of POMC and NPY neurons in the hypothalamus Dev Biol 2011 8 406 416 10.1016/j.ydbio.2010.11.007 21074524

[B5] Bray SJ Notch signalling: a simple pathway becomes complex Nat Rev Mol Cell Biol 2006 8 678 689 10.1038/nrm2009 16921404

[B6] Kawaguchi A Ikawa T Kasukawa T Ueda HR Kurimoto K Saitou M Matsuzaki F Single-cell gene profiling defines differential progenitor subclasses in mammalian neurogenesis Development 2008 8 3113 3124 10.1242/dev.022616 18725516

[B7] Fisher AL Ohsako S Caudy M The WRPW motif of the hairy-related basic helix-loop-helix repressor proteins acts as a 4-amino-acid transcription repression and protein-protein interaction domain Mol Cell Biol 1996 8 2670 2677 8649374 10.1128/mcb.16.6.2670 PMC231257

[B8] Ohtsuka T Ishibashi M Gradwohl G Nakanishi S Guillemot F Kageyama R Hes1 and Hes5 as notch effectors in mammalian neuronal differentiation Embo J 1999 8 2196 2207 10.1093/emboj/18.8.2196 10205173 PMC1171303

[B9] Henrique D Hirsinger E Adam J Le Roux I Pourquie O Ish-Horowicz D Lewis J Maintenance of neuroepithelial progenitor cells by Delta-Notch signalling in the embryonic chick retina Curr Biol 1997 8 661 670 10.1016/S0960-9822(06)00293-4 9285721

[B10] Mizutani K Saito T Progenitors resume generating neurons after temporary inhibition of neurogenesis by Notch activation in the mammalian cerebral cortex Development 2005 8 1295 1304 10.1242/dev.01693 15750183

[B11] Lutolf S Radtke F Aguet M Suter U Taylor V Notch1 is required for neuronal and glial differentiation in the cerebellum Development 2002 8 373 385 11807030 10.1242/dev.129.2.373

[B12] Hirata H Tomita K Bessho Y Kageyama R Hes1 and Hes3 regulate maintenance of the isthmic organizer and development of the mid/hindbrain Embo J 2001 8 4454 4466 10.1093/emboj/20.16.4454 11500373 PMC125583

[B13] Bertrand N Castro DS Guillemot F Proneural genes and the specification of neural cell types Nat Rev Neurosci 2002 8 517 530 10.1038/nrn874 12094208

[B14] Nelson BR Hartman BH Georgi SA Lan MS Reh TA Transient inactivation of Notch signaling synchronizes differentiation of neural progenitor cells Dev Biol 2007 8 479 498 10.1016/j.ydbio.2007.01.001 17280659 PMC1979095

[B15] Shimojo H Ohtsuka T Kageyama R Dynamic expression of notch signaling genes in neural stem/progenitor cells Front Neurosci 2011 8 78 21716644 10.3389/fnins.2011.00078 PMC3116140

[B16] Aujla PK Naratadam GT Xu L Raetzman LT Notch/Rbpjkappa signaling regulates progenitor maintenance and differentiation of hypothalamic arcuate neurons Development 2013 8 3511 3521 10.1242/dev.098681 23884446 PMC3742139

[B17] Kageyama R Ohtsuka T Shimojo H Imayoshi I Dynamic Notch signaling in neural progenitor cells and a revised view of lateral inhibition Nat Neurosci 2008 8 1247 1251 10.1038/nn.2208 18956012

[B18] Hatakeyama J Bessho Y Katoh K Ookawara S Fujioka M Guillemot F Kageyama R Hes genes regulate size, shape and histogenesis of the nervous system by control of the timing of neural stem cell differentiation Development 2004 8 5539 5550 10.1242/dev.01436 15496443

[B19] Pera EM Kessel M Demarcation of ventral territories by the homeobox gene NKX2.1 during early chick development Dev Genes Evol 1998 8 168 171 10.1007/s004270050170 9601992

[B20] Ware M Schubert FR Development of the early axon scaffold in the rostral brain of the chick embryo J Anat 2011 8 203 216 10.1111/j.1469-7580.2011.01389.x 21599661 PMC3162240

[B21] Li X Chiang HI Zhu J Dowd SE Zhou H Characterization of a newly developed chicken 44 K Agilent microarray BMC Genomics 2008 8 60 10.1186/1471-2164-9-60 18237426 PMC2262898

[B22] Abello G Khatri S Giraldez F Alsina B Early regionalization of the otic placode and its regulation by the Notch signaling pathway Mech Dev 2007 8 631 645 10.1016/j.mod.2007.04.002 17532192

[B23] Ross DA Kadesch T Consequences of Notch-mediated induction of Jagged1 Exp Cell Res 2004 8 173 182 10.1016/j.yexcr.2004.02.003 15149848

[B24] Rebeiz M Miller SW Posakony JW Notch regulates numb: integration of conditional and autonomous cell fate specification Development 2011 8 215 225 10.1242/dev.050161 21148185 PMC3005598

[B25] Pirot P van Grunsven LA Marine JC Huylebroeck D Bellefroid EJ Direct regulation of the Nrarp gene promoter by the Notch signaling pathway Biochem Biophys Res Commun 2004 8 526 534 10.1016/j.bbrc.2004.07.157 15325262

[B26] Johnston SH Rauskolb C Wilson R Prabhakaran B Irvine KD Vogt TF A family of mammalian Fringe genes implicated in boundary determination and the Notch pathway Development 1997 8 2245 2254 9187150 10.1242/dev.124.11.2245

[B27] Ricano-Cornejo I Altick AL Garcia-Pena CM Nural HF Echevarria D Miquelajauregui A Mastick GS Varela-Echavarria A Slit-Robo signals regulate pioneer axon pathfinding of the tract of the postoptic commissure in the mammalian forebrain J Neurosci Res 2011 8 1531 1541 10.1002/jnr.22684 21688288 PMC4128405

[B28] Stein R Mori N Matthews K Lo LC Anderson DJ The NGF-inducible SCG10 mRNA encodes a novel membrane-bound protein present in growth cones and abundant in developing neurons Neuron 1988 8 463 476 10.1016/0896-6273(88)90177-8 3272176

[B29] de la Pompa JL Wakeham A Correia KM Samper E Brown S Aguilera RJ Nakano T Honjo T Mak TW Rossant J Conlon RA Conservation of the Notch signalling pathway in mammalian neurogenesis Development 1997 8 1139 1148 9102301 10.1242/dev.124.6.1139

[B30] Pape M Doxakis E Reiff T Duong CV Davies A Geissen M Rohrer H A function for the calponin family member NP25 in neurite outgrowth Dev Biol 2008 8 434 443 10.1016/j.ydbio.2008.07.001 18652818

[B31] Helle KB Corti A Metz-Boutigue MH Tota B The endocrine role for chromogranin A: a prohormone for peptides with regulatory properties Cell Mol Life Sci 2007 8 2863 2886 10.1007/s00018-007-7254-0 17717629 PMC11135995

[B32] Sakuta H Suzuki R Takahashi H Kato A Shintani T Iemura S Yamamoto TS Ueno N Noda M Ventroptin: a BMP-4 antagonist expressed in a double-gradient pattern in the retina Science 2001 8 111 115 10.1126/science.1058379 11441185

[B33] Ohyama K Ellis P Kimura S Placzek M Directed differentiation of neural cells to hypothalamic dopaminergic neurons Development 2005 8 5185 5197 10.1242/dev.02094 16284116

[B34] Gohlke JM Armant O Parham FM Smith MV Zimmer C Castro DS Nguyen L Parker JS Gradwohl G Portier CJ Guillemot F Characterization of the proneural gene regulatory network during mouse telencephalon development BMC Biol 2008 8 15 10.1186/1741-7007-6-15 18377642 PMC2330019

[B35] Pacary E Heng J Azzarelli R Riou P Castro D Lebel-Potter M Parras C Bell DM Ridley AJ Parsons M Guillemot F Proneural transcription factors regulate different steps of cortical neuron migration through Rnd-mediated inhibition of RhoA signaling Neuron 2011 8 1069 1084 10.1016/j.neuron.2011.02.018 21435554 PMC3383999

[B36] Cau E Gradwohl G Casarosa S Kageyama R Guillemot F Hes genes regulate sequential stages of neurogenesis in the olfactory epithelium Development 2000 8 2323 2332 10804175 10.1242/dev.127.11.2323

[B37] Cau E Gradwohl G Fode C Guillemot F Mash1 activates a cascade of bHLH regulators in olfactory neuron progenitors Development 1997 8 1611 1621 9108377 10.1242/dev.124.8.1611

[B38] Fior R Henrique D A novel hes5/hes6 circuitry of negative regulation controls Notch activity during neurogenesis Developmental biology 2005 8 318 333 10.1016/j.ydbio.2005.03.017 15893982

[B39] Ishibashi M Ang SL Shiota K Nakanishi S Kageyama R Guillemot F Targeted disruption of mammalian hairy and Enhancer of split homolog-1 (HES-1) leads to up-regulation of neural helix-loop-helix factors, premature neurogenesis, and severe neural tube defects Genes Dev 1995 8 3136 3148 10.1101/gad.9.24.3136 8543157

[B40] McNay DE Pelling M Claxton S Guillemot F Ang SL Mash1 is required for generic and subtype differentiation of hypothalamic neuroendocrine cells Mol Endocrinol 2006 8 1623 1632 16469766 10.1210/me.2005-0518

[B41] Zhu X Zhang J Tollkuhn J Ohsawa R Bresnick EH Guillemot F Kageyama R Rosenfeld MG Sustained Notch signaling in progenitors is required for sequential emergence of distinct cell lineages during organogenesis Genes Dev 2006 8 2739 2753 10.1101/gad.1444706 17015435 PMC1578699

[B42] Tateya T Imayoshi I Tateya I Ito J Kageyama R Cooperative functions of Hes/Hey genes in auditory hair cell and supporting cell development Dev Biol 2011 8 329 340 10.1016/j.ydbio.2011.01.038 21300049

[B43] Haddon C Jiang YJ Smithers L Lewis J Delta-Notch signalling and the patterning of sensory cell differentiation in the zebrafish ear: evidence from the mind bomb mutant Development 1998 8 4637 4644 9806913 10.1242/dev.125.23.4637

[B44] Kokubo H Miyagawa-Tomita S Nakazawa M Saga Y Johnson RL Mouse hesr1 and hesr2 genes are redundantly required to mediate Notch signaling in the developing cardiovascular system Dev Biol 2005 8 301 309 10.1016/j.ydbio.2004.10.025 15680351

[B45] Przemeck GK Heinzmann U Beckers J de Hrabe Angelis M Node and midline defects are associated with left-right development in Delta1 mutant embryos Development 2003 8 3 13 10.1242/dev.00176 12441287

[B46] Swiatek PJ Lindsell CE del Amo FF Weinmaster G Gridley T Notch1 is essential for postimplantation development in mice Genes Dev 1994 8 707 719 10.1101/gad.8.6.707 7926761

[B47] Puelles L Kuwana E Puelles E Bulfone A Shimamura K Keleher J Smiga S Rubenstein JL Pallial and subpallial derivatives in the embryonic chick and mouse telencephalon, traced by the expression of the genes Dlx-2, Emx-1, Nkx-2.1, Pax-6, and Tbr-1 J Comp Neurol 2000 8 409 438 10.1002/1096-9861(20000828)424:3<409::AID-CNE3>3.0.CO;2-7 10906711

[B48] Mori K Muto Y Kokuzawa J Yoshioka T Yoshimura S Iwama T Okano Y Sakai N Neuronal protein NP25 interacts with F-actin Neurosci Res 2004 8 439 446 10.1016/j.neures.2003.12.012 15041197

[B49] Mahapatra NR Taupenot L Courel M Mahata SK O'Connor DT The trans-Golgi proteins SCLIP and SCG10 interact with chromogranin A to regulate neuroendocrine secretion Biochemistry 2008 8 7167 7178 10.1021/bi7019996 18549247 PMC2576284

[B50] Castro DS Martynoga B Parras C Ramesh V Pacary E Johnston C Drechsel D Lebel-Potter M Garcia LG Hunt C Dolle D Bithell A Ettwiller L Buckley N Guillemot F A novel function of the proneural factor Ascl1 in progenitor proliferation identified by genome-wide characterization of its targets Genes Dev 2011 8 930 945 10.1101/gad.627811 21536733 PMC3084027

[B51] Ishii J Sato H Sakaeda M Shishido-Hara Y Hiramatsu C Kamma H Shimoyamada H Fujiwara M Endo T Aoki I Yazawa T POU domain transcription factor BRN2 is crucial for expression of ASCL1, ND1 and neuroendocrine marker molecules and cell growth in small cell lung cancer Pathol Int 2013 8 158 168 10.1111/pin.12042 23530560

[B52] O'Rahilly S Human genetics illuminates the paths to metabolic disease Nature 2009 8 307 314 10.1038/nature08532 19924209

[B53] Hamburger V Hamilton HL A series of normal stages in the development of the chick. 1951 Dev Dyn 1992 8 231 272 10.1002/aja.1001950404 1304821

[B54] Dupé V Rochard L Mercier S Le Petillon Y Gicquel I Bendavid C Bourrouillou G Kini U Thauvin-Robinet C Bohan TP Odent S Dubourg C David V NOTCH, a new signaling pathway implicated in holoprosencephaly Hum Mol Genet 2011 8 1122 1131 10.1093/hmg/ddq556 21196490 PMC3390777

[B55] Geling A Steiner H Willem M Bally-Cuif L Haass C A gamma-secretase inhibitor blocks Notch signaling in vivo and causes a severe neurogenic phenotype in zebrafish EMBO Rep 2002 8 688 694 10.1093/embo-reports/kvf124 12101103 PMC1084181

[B56] Chapman SC Schubert FR Schoenwolf GC Lumsden A Analysis of spatial and temporal gene expression patterns in blastula and gastrula stage chick embryos Dev Biol 2002 8 187 199 10.1006/dbio.2002.0641 11969265

[B57] Lumsden A Keynes R Segmental patterns of neuronal development in the chick hindbrain Nature 1989 8 424 428 10.1038/337424a0 2644541

[B58] Cartharius K Frech K Grote K Klocke B Haltmeier M Klingenhoff A Frisch M Bayerlein M Werner T MatInspector and beyond: promoter analysis based on transcription factor binding sites Bioinformatics 2005 8 2933 2942 10.1093/bioinformatics/bti473 15860560

